# Leveraging Quality Improvement Tools to Improve Administration of First-line Surgical Antibiotic Prophylaxis in Patients Labeled as Penicillin Allergic

**DOI:** 10.1097/pq9.0000000000000794

**Published:** 2025-01-23

**Authors:** Madeline Mock, David Morris, Jessica Foley, Mellissa Mahabee, J. Michael Klatte, Beth Williams, Daniel Robie

**Affiliations:** From the * Department of Quality Improvement, Dayton Children’s Hospital, Dayton, Ohio; † Division of Allergy and Immunology, Dayton Children’s Hospital, Dayton, Ohio; ‡ Department of Pediatrics, Wright State University Boonshoft School of Medicine, Dayton, Ohio; § Department of Pharmacy, Dayton Children’s Hospital, Dayton, Ohio; ¶ Division of Critical Care, Dayton Children’s Hospital, Dayton, Ohio; ∥ Division of Infectious Disease, Dayton Children’s Hospital, Dayton, Ohio; ** Division of Pediatric Surgery, Dayton Children’s Hospital, Dayton, Ohio.

## Abstract

**Introduction:**

A reported penicillin allergy reduces the likelihood that the patient will receive first-line surgical antibiotic prophylaxis (SAP), which can increase the risk of developing a surgical site infection (SSI). This project aimed to increase the use of first-line SAP agents in orthopedic and pediatric surgery patients with a reported penicillin allergy.

**Methods:**

The Institute for Healthcare Improvement quality improvement methodology was followed. Key drivers included patient and family awareness of true penicillin allergies, standardization for ordering antibiotics, staff buy-in, electronic medical record utilization, and staff comfort with ordering first-line SAP. Initial plan-do-study-act cycles focused on provider education. Subsequent plan-do-study-act cycles focused on the antibiotic delivery process, antibiotic selection, screening tool development for severe delayed hypersensitivity reactions, education, and data transparency. The outcome measure was the percentage of orthopedic and pediatric surgery patients with a reported penicillin allergy that received first-line SAP per month.

**Results:**

Since the start of the project in December 2022, there were 2 statistically significant changes in the outcome measure’s mean, shifting the mean from 25% to 84% in orthopedic and pediatric surgery patients with a reported penicillin allergy who received first-line SAP. There were no adverse medication reactions and no statistically significant change in SSIs.

**Conclusions:**

The mean has been at 84% for 9 months showing a sustainable process and culture change regarding first-line SAP usage for orthopedic and pediatric surgery patients. This was achieved through targeting the antibiotic delivery processes without relying on hard stops within the medical record.

## INTRODUCTION

### Problem Description

In patients reporting a penicillin allergy, 10% or less have a true allergy.^1^ However, due to cross-reactivity concerns, these patients have avoided cephalosporins. Several authors report that the incidence of surgical site infections (SSIs) is increased in patients reporting a penicillin allergy.^1–7^ It is believed this is related to avoiding first-line surgical antibiotic prophylaxis (SAP) in these patients. As part of ongoing efforts to reduce SSIs, an opportunity to improve the use of first-line SAP in patients with a reported penicillin allergy was identified. We hypothesized that increasing the use of first-line SAP in patients with a reported penicillin allergy could be achieved through interventions aimed at the antibiotic ordering and delivery process.

### Available Knowledge

Approximately 2%–4% of patients undergoing surgery will experience an SSI.^8^ The use of SAP for selected surgical reduces SSIs, and their use has become the standard of care and a component of all SSI prevention bundles. These bundles also require the antibiotics be delivered within 60 minutes before the surgical incision. Beta-lactam antibiotics (most commonly cephalosporins) are the first-line choice for prophylaxis and are more effective in preventing SSIs than non-beta-lactam antibiotics.^5,6^ Patients with a reported penicillin allergy are less likely to receive cephalosporins, and multiple reports have shown an increase in SSIs in this population.^1–7^

Multiple authors have published their experiences with increasing the use of cephalosporins in this population.^9–11^ Macy removed an automated electronic medical records (EMRs) alert warning against prescription of cephalosporins for all patients with penicillin allergies.^9^ Grant provided education for physicians and modified preprinted presurgical order sets.^10^ Isserman et al^11^ provided education to anesthesia providers, an SAP reference guide, and postprescription email reminders. All 3 studies observed sizable increases in first-line cephalosporin usage.

Published quality improvement (QI) initiatives have not only focused on prescribers but have also incorporated direct patient input and allergy clarification.^1,12–15^ Sexton et al^12^ and Goh et al^13^ administered preoperative questionnaires to patients with a reported penicillin allergy to allow for allergy risk stratification.^13^ When questionnaire utilization was combined with education and creation of decision support algorithms, first-line SAP increased by 10% (84%–94%) and 70% (11%–80%), respectively. Additionally, plan-do-study-act (PDSA) approaches at 2 children’s hospitals integrated preoperative telephone interviews with caregivers and primary care providers for allergy clarification.^14,15^

A more recent study by He et al^14^ interacted with surgeons and residents via emails when cases were identified in advance of surgery in patients with a penicillin allergy. They also sent emails when noncompliance was identified postoperatively. Rates for “appropriate” SAP for penicillin-allergic patients increased from 34.5% to 88.5%.^14^ They included patients as receiving “appropriate” SAP even if they screened positive using their decision support algorithm and thus received second-line SAP, whereas we only included those patients who received first-line SAP.

### Rationale

Historically, cephalosporins have been avoided in patients with a reported penicillin allergy due to concerns related to immunologic cross-reactivity.^10^ Cefazolin, a first-generation cephalosporin, is recommended as first-line SAP for numerous operative procedures because of activity against skin flora such as methicillin-susceptible *Staphylococcus aureus*, and various species of streptococci.^16^ For individuals with a penicillin allergy, immunologic cross-reactivity to cephalosporins is mediated primarily by structural similarities of the R1 side chains.^12,17^ The R1 side chain of cefazolin and penicillin antibiotics do not possess any structural similarities, and thus cross-reactivity resulting in anaphylaxis, is rare.^1,17–19^ This is also the case for commonly used antibiotics such as cefuroxime (second generation) and ceftriaxone (third generation).^17^ Relative to cefazolin, second-line agents such as clindamycin and vancomycin provide coverage against a broader spectrum of organisms.^20^ Utilization of these broader-spectrum agents can promote antibiotic resistance, increase adverse events, and their use as second-line SAP agents is associated with an increased risk of SSIs in comparison to first-line antibiotics.^14^ Of note, the cross-reactivity of cephalosporins in patients with a history of T cell–mediated delayed hypersensitivity reactions such as Steven-Johnson syndrome, toxic epidermal necrosis, and vasculitis is less clear. Such reactions can recognize the whole or part of the beta-lactam molecule, and therefore, cephalosporins are typically avoided in these patients.^21^ Finally, though there have been reported cases of anaphylaxis or anaphylactic shock to cephalosporins in adults with a history of penicillin allergy, we have not identified any reported cases in children.^22,23^

### Aim

This QI project aimed to increase the use of first-line SAP in orthopedic and pediatric surgery patients with a reported penicillin allergy from a baseline of 25% to a goal of 75% over 15 months.

## METHODS

### Context

On average, the orthopedic division performs 1,500 cases per year (including spinal fusions, sports injuries, trauma, and hand surgery) and the pediatric surgery division performs 1,600 per year (including congenital anomalies, gastrostomy, central lines, tumor resection, appendectomy). We do not perform joint replacement surgery or transplant surgery. This project encompassed all class 1 (clean) and 2 (clean-contaminated) cases performed by these specialties for which SAP was used. We also included patients with appendicitis who received therapeutic antibiotics (eg, ceftriaxone) at the time of surgery. We excluded all other class 3 (infected) and class 4 (dirty) cases. This exclusion resulted in the elimination of 5 cases.

The decision to start with 2 surgical divisions was based on the small test of change approach to QI and to identify any roadblocks to implementation. In addition, these were 2 high volume specialties with many cases requiring SAP.

### Interventions

**Fig. 1.**
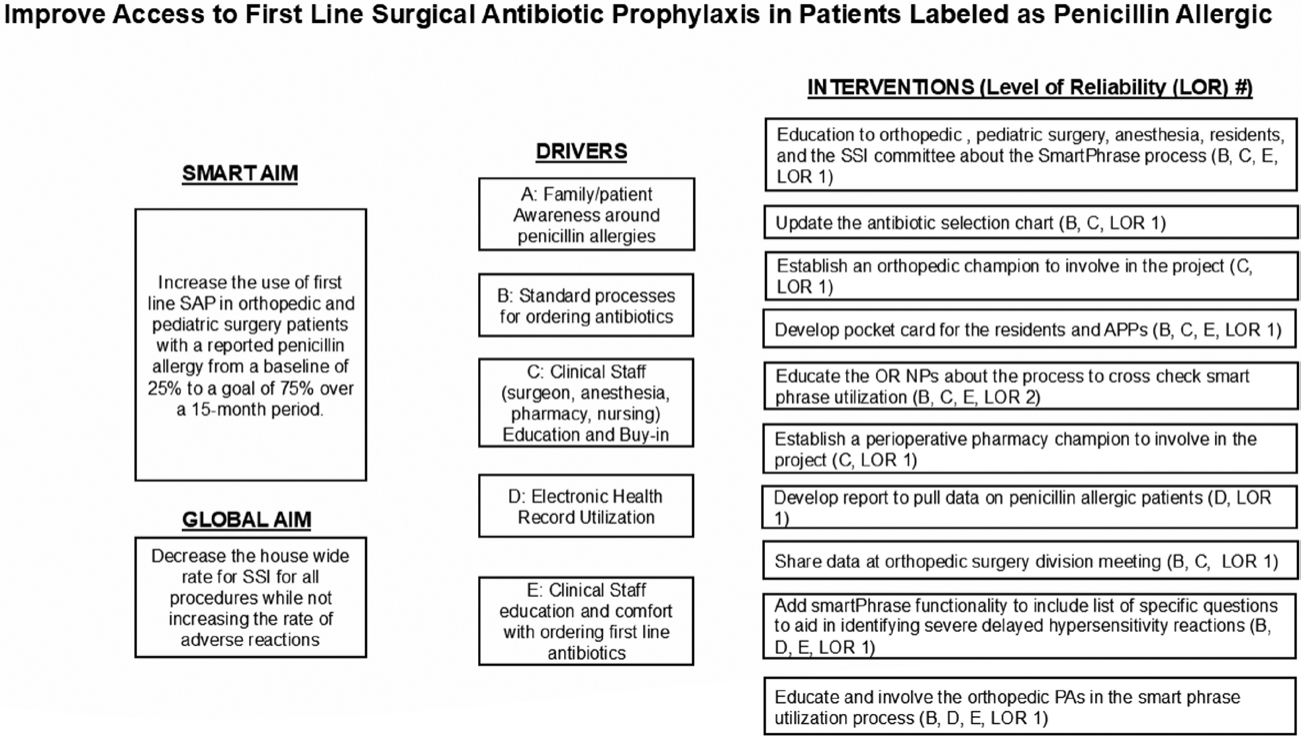
Key driver diagram, illustrating the completed interventions for the project. Each driver is assigned a letter that is used to map the relationship to each intervention (shown in parenthesis). The level of reliability indicates the ability of the intervention to operate without failure.

The team consisted of the Surgeon in Chief, an allergy physician, the medical director of quality, an infectious disease pharmacist, an infectious disease physician, and a QI consultant. The team followed the Institute for Healthcare Improvement methodology.^24^ The key driver diagram is shown in Figure 1.

#### Antibiotic Selection Chart

The hospital maintains a prophylactic antibiotic selection chart accessible via hyperlink within the EMR. The SAP recommendations by surgical subspecialty and type of surgery were determined based on guidance from the American College of Surgeons National Surgical Quality Improvement Program (ACS NSQIP-P) SAP Utilization Compliance Guidelines. In addition, we asked division chiefs if they preferred alternative antibiotics based on local experience. This rarely differed from the ACS consensus guidelines but improved buy-in by the providers.

#### Screening Tool Development

**Fig. 2.**
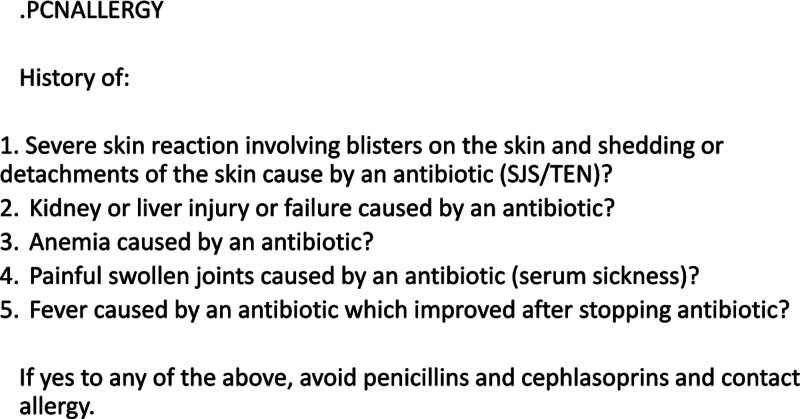
Penicillin allergy critical questions. The critical questions were adapted from the work of Kuruvilla et al.^25^ The critical questions screen patients with a reported penicillin allergy for the presence of an SDHR to aid in appropriate antibiotic selection.

We created a set of questions to screen patients with a reported penicillin allergy for the presence of severe delayed hypersensitivity reactions (SDHRs). These questions were adapted from published work by Kuruvilla et al.^25^ The questions were developed into a SmartPhrase (text that can be pulled into the EMR by placing a dot in front of the phrase) for ease of use (Fig. 2).^26^

When a penicillin allergy is reported, the provider enters the SmartPhrase into the allergy section of the EMR based on patient responses. If a patient answers “yes” to any of the 5 questions, they do not receive a cephalosporin and are referred to allergy for testing. If a patient answers “no” to all questions, the patient is considered not to have a severe penicillin allergy and can receive first-line SAP.

#### Provider Education

The team presented an overview of the literature, QI methodology, and the SmartPhrase to the pediatric surgery, orthopedic surgery and anesthesiology divisions and the hospital’s SSIs committee. It was subsequently given to the perioperative staff and the operating room nurse practitioners (OR NPs). Additional rounds of re-education were given over the subsequent nine months.

#### Antibiotic Prophylaxis Delivery Process

**Fig. 3.**
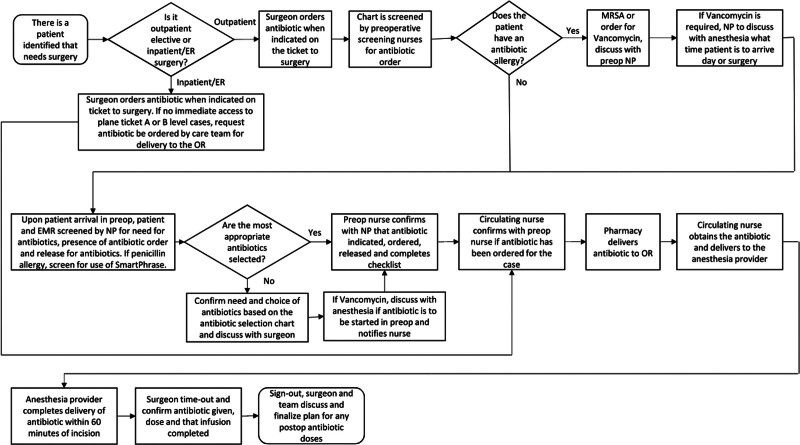
Antibiotic prophylaxis delivery process map. The process map uses a square with rounded corners to denote the beginning and end of the process map for antibiotic prophylaxis delivery for surgery patients. The diamonds are used to indicate a decision. The squares are used for process steps. The arrows show the process flow.

A workflow was developed that empowered the OR NPs to engage in correct SAP selection (Fig. 3). This most commonly occurs when the patient arrives in the perioperative area. At that time, the EMR is reviewed and if a penicillin allergy is documented, the NP screens for SDHRs using the SmartPhrase. If necessary, the NP then reaches out to the surgeon to ensure first-line SAP has been ordered based on the results of the screening. The residents are responsible for placing the ticket to surgery which includes appropriate SAP selection.

#### Data Transparency

The team compared the orthopedic and pediatric surgery compliance rates each month for administering first-line SAP agents to patients with a reported penicillin allergy. The comparisons were shared via a control chart with the respective divisions at the department of surgery meeting, the quarterly surgical case review conferences and other times throughout the project to stimulate positive competition toward the desired behavior.

#### Resident Cards

At the start of their respective months, we educated resident surgeons on their pediatric surgery and pediatric orthopedic surgery rotations about the project and the process for evaluating patients with reported penicillin allergy. We developed a 3 × 5-inch laminated pocket card containing the SmartPhrase and instructions for use in the EMR for the residents.

### Study of the Interventions

The hospital’s Clinical Informatics Department developed a PowerBI (Microsoft) report to identify all orthopedic and pediatric surgery patients with a documented penicillin allergy in the EMR. This report was used to gather baseline data from January to November 2022 for first-line SAP usage. Subsequent data was collected monthly through the remainder of the project. Each intervention was tested as a unique PDSA cycle. This allowed the team to determine the impact of each intervention and adapt subsequent interventions based on lessons learned.^24^ This project followed SQUIRE 2.0 reporting guidelines.^27^

### Measures

The outcome measure analyzed was the percentage of orthopedic and pediatric surgery patients with a reported penicillin allergy who received first-line SAP. For this measure, patients who answered “yes” to the critical questions were excluded from the data analysis because they were ineligible to receive first-line SAP. There was a total of 11 patients excluded for this reason.

The process measure analyzed was the SmartPhrase utilization rate. The patients who answered “yes” to any of the critical questions were included in this data analysis, as it captured the utilization of the screening tool in the relevant patient population.

To ensure accurate determination of compliance with first-line SAP and use of the SmartPhrase, the raw monthly data was reviewed by the team for accuracy and to eliminate cases where there was a “yes” response to the critical questions.

As a balancing safety measure, adverse medication reactions to antibiotics were tracked following the launch of the SmartPhrase in April 2023. The information was gathered from anesthesiology personnel through an EMR reporting tool utilized to track anesthesia complications, including reactions to antibiotics. This is done through the Pediatric Anesthesia Quality Improvement Initiative called Wake Up Safe.^28^

In addition, SSI rates were monitored for any changes during the study period. SSIs are identified through various sources including NSQIP-P, surgical morbidity and mortality conferences, hospital-acquired condition team, and infection control. SSI determination is made based on established definitions provided by NSQIP-P and the National Healthcare Safety Network. All SSIs are reviewed at the multidisciplinary SSI committee meetings and include a standardized drilldown to identify any preoperative, operative, and postoperative contributing factors.

### Analysis

Data were displayed in a control chart over time to determine of special cause variation (any point outside the control limits), common cause variation, and statistically significant shifts. The team followed the shift rule which states there must be 8 consecutive points either above or below the mean. The control charts were annotated with the interventions to determine each intervention’s effectiveness.^29^

### Ethical Considerations

The project’s aim provided a benefit in risk reduction of postoperative SSI development. It was critical to ensure no increase in allergic reactions by using cephalosporins as this directly addressed the medical ethics principle of nonmaleficence. The team took the precautionary step of screening for SDHRs and avoiding use in those patients. The multidisciplinary team allowed for robust discussion and informed decisions to ensure the project was safe.

## RESULTS

Baseline data showed that 25% of orthopedic and pediatric surgery patients with a reported penicillin allergy received first-line SAP. From the start of interventions in February 2023 through April 2024, there have been 224 patient encounters that met the criteria for this project. That represents 5.9 % of the total cases performed by the 2 surgical divisions during that timeframe. Of those encounters, 140 were from orthopedic surgery and 84 were from pediatric surgery.

**Fig. 4.**
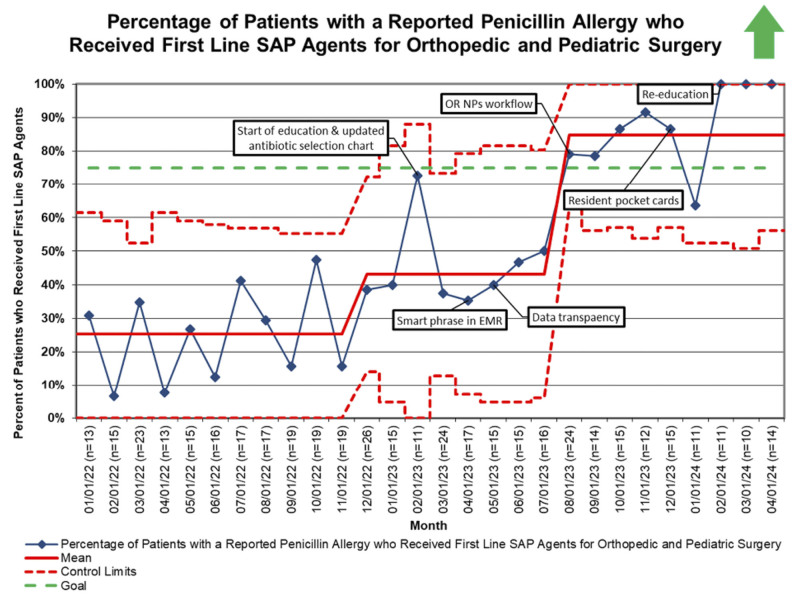
Orthopedic and pediatric surgery first-line SAP usage in patients with a reported penicillin allergy. A p-chart was used to display the percentage of orthopedic and pediatric surgery patients with a reported penicillin allergy that received first-line SAP. The upper and lower control limits are approximately ± 3σ from the mean and vary based on the sample size. Patients who screened positive for SDHR are excluded from these data.

In February 2023, the team observed an increase in compliance that we attributed to more awareness on the topic. The first PDSA focused on the education of all necessary stakeholders. The second PDSA cycle, the SmartPhrase, went live in the EMR in April 2023 and continued to help increase compliance. The third PDSA cycle involved sharing compliance data amongst the Orthopedic and Pediatric Surgery divisions. While working on these PDSAs, the first statistically significant shift in the data occurred in December 2022, bringing the mean to 43% (Fig. 4). The fourth PDSA cycle created a new antibiotic delivery process empowering the OR NPs. A second statistically significant shift in August 2023 brought the mean to 84%, above the goal of 75% (Fig. 4).

**Fig. 5.**
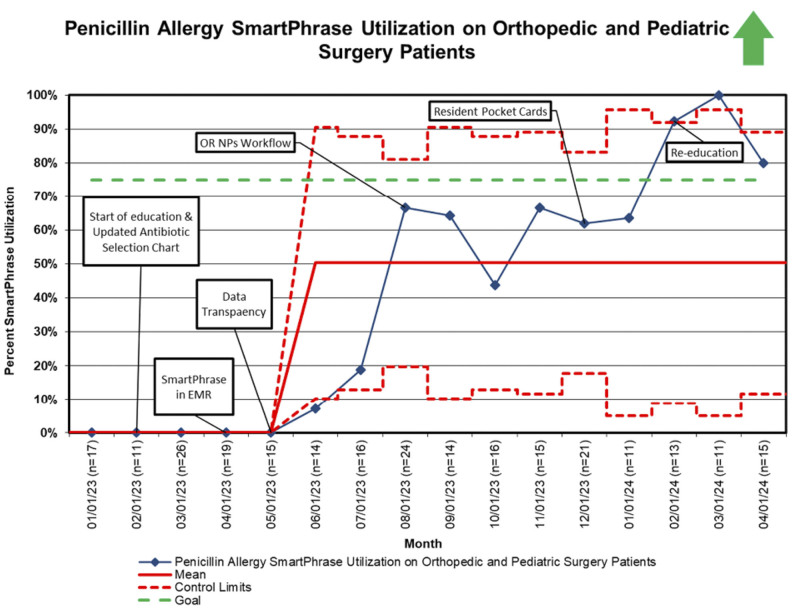
Usage of SmartPhrase for identifying SDHRs. A p-chart was used to display the usage of the SmartPhrase in orthopedic and pediatric surgery patients with a reported penicillin allergy. The SmartPhrase contains the penicillin allergy critical questions. Patients who screened positive for SDHR are included in these data.

Once the SmartPhrase went live in the EMR, we tracked utilization to ensure providers were following the evidence-based recommendations to aid in appropriate antibiotic selection (Fig. 5). The mean shifted in June 2023 from 0% to 50%, below our goal of 75%.

**Fig. 6.**
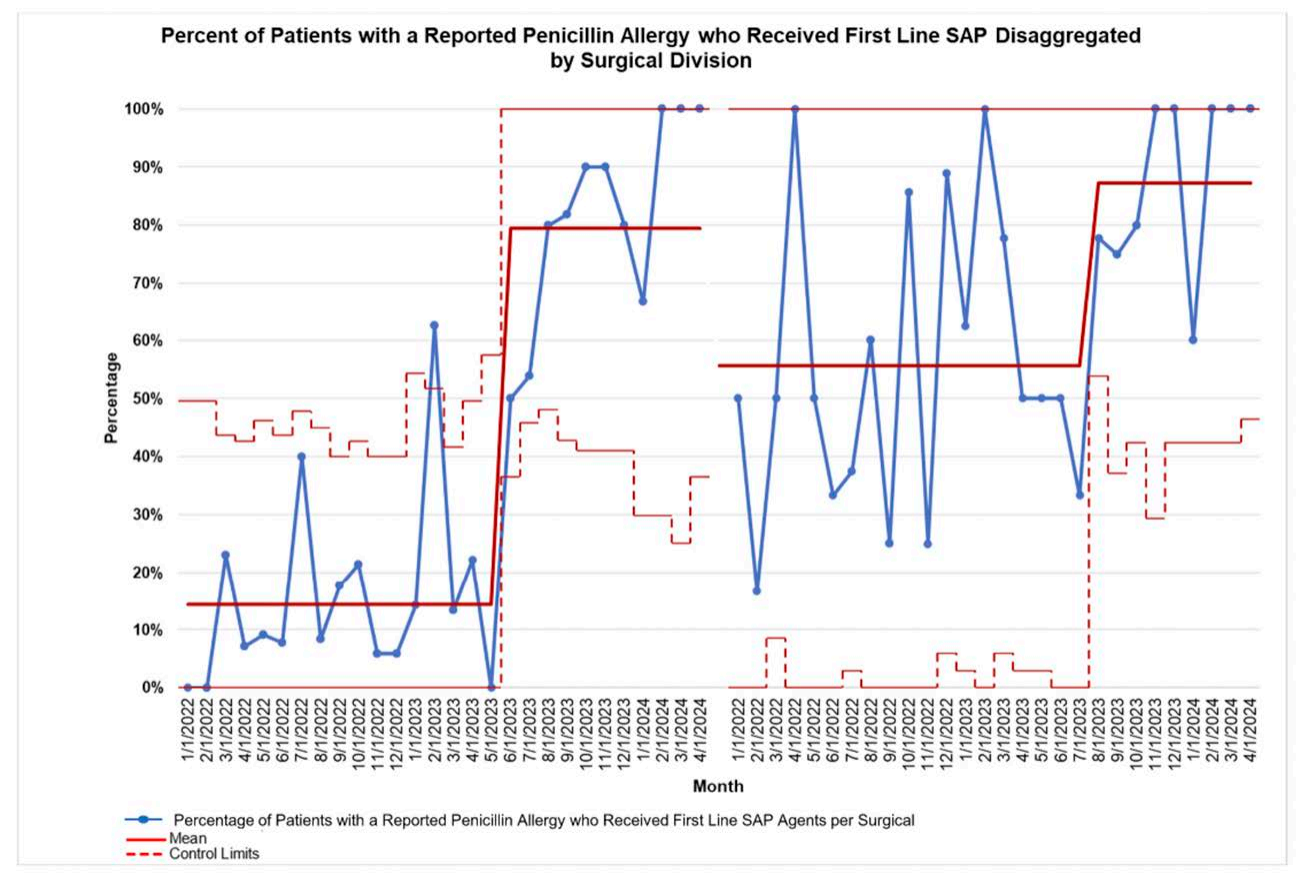
Orthopedic and pediatric surgery comparison for first-line SAP usage in patients with a reported penicillin allergy. The drilldown pathway was followed to disaggregate the data into the 2 surgical divisions of interest. The data displayed on the p-chart is sequenced by surgical division. The lefthand side displays the compliance for orthopedic surgery and the righthand side displays the compliance for pediatric surgery. The dashed red line is the upper and low control limits which are approximately ± 3σ from the mean and vary based on the sample size. The solid line is the mean. The total number of surgical encounters from orthopedic surgery is 140. The total number of surgical encounters from pediatric surgery is 84.

Figure 6 illustrates that the intervention focused on sharing data between divisions. The new antibiotic delivery process helped close the care gap in utilizing first-line SAP in patients with a reported penicillin allergy. Following these 2 interventions, orthopedic surgery has a compliance of 79%, similar to pediatric surgery, which has a compliance of 87%. Since the launch of the SmartPhrase in April 2023, there have been no reported adverse reactions to antibiotics.

We did see a gradual decrease in institutional SSI rates during the study. However, we caution interpretation of these data as SSI rates depend on capture, and we have continued to expand our sources of SSI reporting. During the study period, our NSQIP-P SSI rates remained “as expected” in all models but we did see a slight trend in increasing infections rates overall. We believe this is best explained by our improved capture of SSIs by our trained chart abstractors.

## DISCUSSION

### Summary

We successfully used a multifaceted approach to increase the use of first-line SAP from 25% to 84% during the 15-month study period. We have sustained our success since the original article submission and have recently rolled the project out to include all surgical divisions. We did not see any increase in adverse drug reactions with the increased use of cephalosporins during the study. We believe the project’s success was based on appreciation of the antibiotic delivery process and leveraging components of that process to achieve lasting change. The interventions were aimed at education of surgical specialties and ordering providers, easy access to linked SAP guidelines during physician order entry, standardization of allergy documentation with screening for SDHRs, and utilization of perioperative OR NPs to ensure compliance. This global approach was critical to achieving universal buy-in and achieving sustainability. We did not find an apparent in SSI rates during our study. We believe this is explained by the multiple variables that can contribute to the occurrence of an SSI.

### Interpretation

The project’s success mirrors the findings of other groups in achieving the institutional culture changes necessary to increase the use of cephalosporins in patients with a documented penicillin allergy.^9,10,12,13^ Although most groups focus on physician education, considered to have the least long-term impact, we took a more global approach by adding interventions such as leveraging the EMR and empowering nonphysician providers to ensure correct antibiotic selection.^9,10^

Previously, access to penicillin allergy testing has been limited due to the need for allergy referral. Novel programs in multiple settings and by different level providers, including pharmacists, have decreased this barrier.^1,30–33^

It was critical to educate the various physician groups and preoperative nursing staff and address their individual concerns. Previous attitudes indicated a reluctance to prescribe cephalosporins in patients with a penicillin allergy.^33^ More recent literature has indicated the safety of cephalosporins with dissimilar side changes in penicillin allergy, even in the absence of allergy testing.^9,17,19^ One example of this was successfully overcoming provider hesitancy of using cephalosporins in patients with reports of anaphylaxis to penicillin. From the onset, the team adopted a cautious approach to ensure identification and exclusion of patients with SDHRs.

It is known that reducing SSIs improves patient outcomes, reduces lengths of stay, and decreases costs of care. Well-accepted interventions aimed at SSI reduction include the implementation of general and procedure-specific SSI prevention bundles, antibiotic stewardship efforts including appropriate selection and dosing, hand hygiene, and more recently, the use of wound protectors and antibiotic-impregnated sutures.

### Limitations

The project’s scope was limited to 2 surgical divisions within a single children’s hospital so our success may not be generalizable to all surgical specialties.

The process relied on manually inserting the critical questions into the allergy section of the EMR, which could negatively impact sustainability. The team is currently investigating the automating use of the SmartPhrase when documenting a penicillin allergy.

As to validation of the data, the team based our antibiotic selection on consensus guidelines for SAP provided by the ACS. When percentage compliance with first-line SAP was measured each month, the antibiotic choice was cross-checked with the Antibiotic Selection Chart to determine the correctness of choice.

## Conclusions

Using a multifaceted approach, the project succeeded in increasing the use of first-line SAP in patients reporting a penicillin allergy in the 2 divisions of focus. We believe this global approach, which included upfront education of key stakeholders, changes to the EMR including decision support, empowerment of nonphysician providers in the selection of SAP, and data transparency, were key to our success. The team screened for SDHRs and excluded patients for whom it would be unsafe to administer cephalosporin in the face of a penicillin allergy. There were no adverse drug reaction events with the increased use of cephalosporins during the study period. The project has shown sustainability over the short term. Achieving long-lasting success will require further modifications to our EMR to reduce dependency in remembering to screen patients for SDHRs and order first-line SAP.
